# Role of Oxidative Stress in Maternal and Neonatal Diseases

**DOI:** 10.1155/2019/9742764

**Published:** 2019-02-24

**Authors:** Carlo Dani, Fabio Mosca, Diego Gazzolo, Federico Mecacci, Irene Cetin, Giuseppe Buonocore

**Affiliations:** ^1^Division of Neonatology, Careggi University Hospital, Florence, Italy; ^2^Department of Clinical and Experimental Medicine, Research Unit of Histology & Embryology, University of Florence, Florence, Italy; ^3^NICU Fondazione IRCCS Ca' Granda Ospedale Maggiore Policlinico, Milan, Italy; ^4^Department of Clinical Sciences and Community Health, University of Milan, Milan, Italy; ^5^Department of Maternal, Fetal and Neonatal Medicine, C. Arrigo Children's Hospital, Alessandria, Italy; ^6^Department of Health Sciences, University of Florence, Obstetrics and Gynecology, Careggi University Hospital, Florence, Italy; ^7^Department of Obstetrics and Gynecology, Vittore Buzzi Children's Hospital, Università di Milano, Milan, Italy; ^8^Università degli Studi di Siena, Italy

The objective of this special issue is to address correlations between oxidative stress and neonatal diseases. Many papers were submitted, and after a thorough peer review process, eight papers were selected to be included in this special issue. These research works provide relevant insights toward better understanding of pathophysiology of some preterm infants' complications and obesity in pregnancy. We believe that published papers via this special issue introduce readers to the latest advances in the field.

The paper by N. Laforgia et al. discusses the role of oxidative stress in the pathophysiology of congenital malformations. Oxidative stress might disrupt signalling pathways with a causative role in birth defects. New insights in the knowledge of these mechanisms of oxidative stress-related congenital malformations represent the basis of possible clinical applications in screening, prevention, and therapies.

The paper by C. Mandò et al. reports that lipotoxic placental environment changes the mitochondrial function, with excessive production of reactive oxygen species in maternal obesity, with increased inflammation and oxidative stress. In obese pregnancies, maternal glycemia or the maternal nutritional status and lifestyle might affect the pattern of mitochondrial dysfunction and possibly affect pregnancy outcomes.

The paper by C. Poggi and C. Dani presents data supporting the oxidative stress involvement in detrimental pathways activated during neonatal sepsis, eventually leading to organ dysfunction and death. Moreover, they discussed the possible role of antioxidant treatment during neonatal sepsis, including melatonin and pentoxifylline, or novel antioxidant molecules, such as edaravone and endothelin receptor antagonists, which are at present under investigation in animal models.

The paper by S. Perrone et al., details that newborns are particularly susceptible to OS and oxidative damage due to the increased generation of free radicals and the lack of adequate antioxidant protection.

They provide an update on the pathogenesis of the so-called “free radical-related diseases of prematurity,” including retinopathy of prematurity, bronchopulmonary dysplasia, intraventricular hemorrhage, periventricular leukomalacia, and necrotizing enterocolitis.

The paper by A. Aceti et al. examines the role of oxidative stress (OS) in the pathogenesis of neonatal necrotizing enterocolitis and explores potential preventive and therapeutic antioxidant strategies. They discuss the protective effect of maternal milk exploring the so-called “milky way” for reducing oxidative stress by implementing human milk feeding and the risk of developing necrotizing enterocolitis. Other possible prophylactic strategies, such as the use of melatonin and lactoferrin, are discussed.

The paper by Silberstein et al. considers that women with preeclampsia suffer from acute oxidative stress and high lipid oxidation in plasma. Therefore, the authors compared levels of polyphenols and lipid peroxidation in colostrum of nursing mothers with and without preeclampsia. They found that polyphenols were higher and lipid oxidation was lower in colostrum of women with preeclampsia, as if it exists a maternal compensation mechanism that protects the newborn from the stress processes the mother experiences. The paper by S. Negro et al. is aimed at evaluating the predictive role of a panel of oxidative stress biomarkers for the early identification of infants at high risk of HIE and their validation through the correlation with MRI findings. Advanced oxidation protein products (AOPP), nonprotein-bound iron (NPBI), and F2-isoprostanes (F2-IsoPs) were measured. Newborns with severe asphyxia showed higher oxidative stress than those with mild asphyxia at birth. AOPP are significantly associated with the severity of brain injury assessed by MRI, especially in males.

The paper by M. C. Pintus et al. evaluated the possible role of metabolomics in diagnosing bronchopulmonary dysplasia (BPD) in a cohort of preterm infants. They found that the discriminant urinary metabolites were alanine, betaine, trimethylamine-N-oxide, lactate, and glycine. They conclude that utilizing metabolomics, it is possible to detect in the first week of life infants who subsequently developed BPD.

